# Endoscopic negative-pressure treatment: From management of complications to pre-emptive active reflux drainage in abdomino-thoracic esophageal resection—A new safety concept for esophageal surgery

**DOI:** 10.1007/s00104-023-01996-6

**Published:** 2023-12-12

**Authors:** Gunnar Loske, Johannes Müller, Wolfgang Schulze, Burkhard Riefel, Matthias Reeh, Christian Theodor Müller

**Affiliations:** 1 Clinic for General, Visceral, Thoracic and Vascular Surgery, Katholisches Marienkrankenhaus Hamburg, Alfredstr. 9, 22087 Hamburg, Germany; 2 Surgery, Wilhelmsburger Krankenhaus Groß-Sand, Groß-Sand 3, 21107 Hamburg, Germany

**Keywords:** Intraluminal endoscopic vacuum therapy, Anastomosis insufficiency, At-risk anastomosis, Prophylaxis, Prevention, Intraluminale endoskopische Vakuumtherapie, Anastomoseninsuffizienz, Risikoanastomose, Prophylaxe, Prävention

## Abstract

**Introduction:**

Early postoperative reflux (PR) can compromise anastomotic healing after Ivor Lewis esophagectomy (ILE) and poses a risk for aspiration. Anastomotic insufficiency is the most threatening surgical complication. We present the protective method of pre-emptive active reflux drainage (PARD) with simultaneous enteral feeding. We report our experience with this new safety concept in esophageal surgery in a cohort of 43 patients.

**Materials and Methods:**

For PARD we use a double lumen open porous film drainage (dOFD). To create the dOFD, the gastric tube of a Trelumina probe (Freka®Trelumina, Fresenius) is coated with a double-layered open-pore drainage film (Suprasorb®CNP drainage film, Lohmann & Rauscher) over a length of 25 cm. The dOFD is endoscopically inserted into the tubular stomach intraoperatively after completion of the anastomosis. Continuous negative pressure is applied with an electronic pump (−125 mm Hg). The PR is continuously aspirated completely and the stomach and anastomotic region are decompressed. At the same time, nutrition is delivered via an integrated intestinal tube. Depending on the results of the endoscopic control after 5 days, PARD is either continued or terminated.

**Results:**

During the observation period (2017–2023), PARD was used in all patients (*n* = 43) with ILE. The healing rate under PARD was 100% and healing was observed in all anastomoses. No additional endoscopic procedures or surgical revisions of the anastomoses were required. The median duration of PARD was 8 days (range 4–21). We observed problems in the healing of the anastomosis in 20 of 43 patients (47%) for whom we defined endoscopic criteria for at-risk anastomosis.

**Conclusions:**

Our results suggest that PARD has a strong protective effect on anastomotic healing and may reduce the risk of anastomotic insufficiency. The integrated feeding tube of the dOFD allows early postoperative enteral feeding while simultaneously applying negative pressure. PARD appears to prevent the negative consequences of impaired anastomotic healing.

## Abstract (de)

**Einleitung:**

Der früh postoperative Reflux (PR) kann die Anastomosenheilung nach Ivor-Lewis-Ösophagektomie (ILE) beeinträchtigen und stellt ein Risiko für Aspirationen dar. Die Anastomoseninsuffizienz ist die bedrohlichste chirurgische Komplikation.Wir stellen die protektive Methode der pre-emptiven aktiven Refluxdrainage (PARD) mit gleichzeitiger enteraler Ernährung vor. Wir berichten über unsere Erfahrungen mit dem neuen Sicherheitskonzept für die Ösophaguschirurgie an einem Patientenkollektiv von 43 Patienten.

**Materialien und Methoden:**

Zur PARD nutzen wir eine doppellumige offenporige Foliendrainage (dOFD). Zur Herstellung der dOFD wird der gastrale Schenkel einer Trelumina Sonde (Freka®Trelumina, Fresenius) über eine Länge von 25 cm mit einer doppellagigen offenporigen Drainagefolie (Suprasorb®CNP Drainagefolie, Lohmann & Rauscher) beschichtet. Die dOFD wird nach Fertigstellung der Anastomose intraoperativ endoskopisch im Schlauchmagen eingeführt. Es wird ein kontinuierlicher Unterdruck mit einer elektronischen Pumpe angelegt (−125 mm Hg). Der PR wird permanent komplett abgesaugt, der Magen und die Anastomosenregion dekomprimiert. Gleichzeitig wird über eine integrierte Intestinalsonde ernährt. Je nach den Ergebnissen der endoskopischen Kontrolle nach 5 Tagen wird PARD entweder fortgesetzt oder beendet.

**Ergebnisse:**

Im Beobachtungszeitraum (2017–2023) wurde PARD bei allen Patienten (*n* = 43) mit ILE eingesetzt. Die Heilungsrate unter PARD betrug 100 %, bei allen Anastomosen wurde eine Abheilung beobachtet. Es wurden keine zusätzlichen endoskopischen Eingriffe oder chirurgische Revisionen an den Anastomosen erforderlich. Die mediane Dauer der PARD betrug 8 Tage (Spanne 4–21). Bei 20 von 43 Patienten (47 %) sahen wir Probleme in der Anastomosenheilung, für die wir endoskopische Kriterien der Risikoanastomose definierten.

**Schlussfolgerung:**

Unsere Ergebnisse legen nahe, dass PARD einen starken protektiven Effekt auf die Anastomosenheilung hat und das Risiko der Anastomoseninsuffizienz reduzieren kann. Die integrierte Ernährungssonde der dOFD ermöglicht simultan zur Unterdruckausübung die frühpostoperative enterale Ernährung. PARD scheint die negativen Folgen einer gestörten Anastomosenheilung zu verhindern.

## Introduction

Abdomino-thoracic esophageal resection is the surgical treatment option for operable carcinomas of the gastro-esophageal junction (AEG type I–II). Stage-adapted neoadjuvant oncological pre-treatment is undertaken preoperatively. The greatest risk in the postoperative period is a healing disorder of the newly created esophago-gastric anastomosis. Anastomotic insufficiency is the most serious surgical complication following abdomino-thoracic esophagectomy [1]. Due to the intrathoracic site of the anastomosis, patients are at risk not only of local infection but also of septic mediastinitis with high morbidity and mortality.

Various factors are involved in the development of anastomotic insufficiency, and several strategies can be used to prevent this serious complication [2]. Technical surgical prerequisites include an anastomosis that is as tension-free as possible and adequate blood flow. However, it has been shown that even under optimal conditions, such as performing the surgery in a high-volume center, the use of minimally invasive surgical techniques and robot-guided or hybrid surgeries [3], as well as intraoperative monitoring of perfusion by fluorescence [4] etc., there is still a significant risk of anastomotic insufficiency [2]. The incidence is high, with a rate of 25% even in recent studies of individual centers [5, 6].

The complication rate after esophagectomy does not correlate with the number of surgeries performed in a hospital. Competent multidisciplinary perioperative complication management seems to be a decisive factor in obtaining good treatment results when postoperative problems occur [7–10]. Interventional endoscopy is of particular importance here [8].

**Fig. 1:**
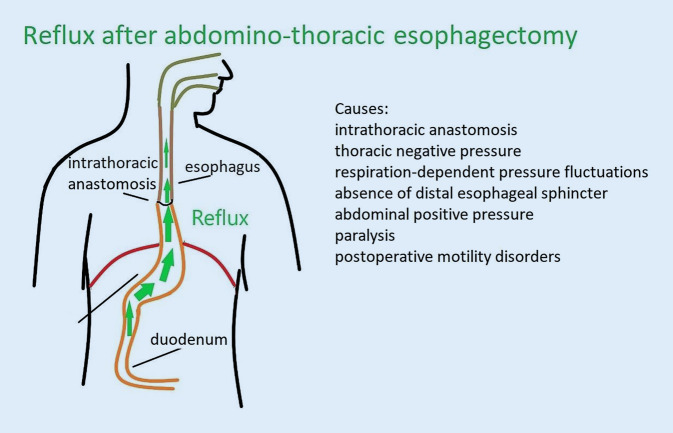
Anatomical situation after abdomino-thoracic esophagectomy and causes of postoperative reflux

The majority of abdomino-thoracic esophagectomies result in reflux due to the altered postoperative anatomy [11, 12]. This is caused by the lack of esophageal closure due to resection of the distal esophageal sphincter, the intrathoracic position of the anastomosis, the pressure gradient between intrathoracic negative pressure and positive abdominal pressure, and postoperative paralysis (Fig. 1; Table 1). Postoperative reflux (PR) can impair anastomotic healing following Ivor–Lewis esophagectomy (ILE) and poses a significant risk of aspiration.

**Table 1:** Causes of postoperative biliary reflux after abdomino-thoracic esophagectomy

Causes of early postoperative reflux after esophagectomy
Intrathoracic anastomosis
Thoracic negative pressure
Respiration-dependent intrathoracic negative-pressure fluctuations
Absence of distal esophageal sphincter
Abdominal positive pressure
Paralysis
Postoperative motility disorders

Routine postoperative endoscopic follow-ups show that despite the insertion of a nasogastric tube (NGT), the anastomotic region is covered with bilious reflux secretions. This means that in the early postoperative healing phase, the anastomosis is permanently exposed to the enzymatically active digestive secretions that exert digestive activity on the newly formed anastomosis. This may jeopardize the local healing process. A green-stained anastomotic wound is typically visible on endoscopy. During endoscopic negative-pressure therapy (ENPT) of anastomotic insufficiencies it is noted that these changes are no longer observed when the reflux is actively suctioned off and that this drainage seems to make a significant contribution to the healing of the anastomosis [13].

Based on the aforementioned clinical findings and our extensive experience in the use of ENPT, we introduced the pre-emptive active reflux drainage (PARD) method with simultaneous enteral nutrition. This method is a further development of intraluminal ENPT for pre-emptive anastomosis prophylaxis. Since 2017, we have been using PARD for all patients undergoing ILE. We have already reported on our experience of using this method on 24 patients [14].

In this article, we report on the use of PARD in a larger cohort of 43 patients.

## Materials and methods

Included in our retrospective observational study were all patients who underwent ILE at the Marienkrankenhaus during the study period between November 2017 and April 2023. Since November 2017, PARD has been an integral part of our treatment approach.

**Fig. 2:**
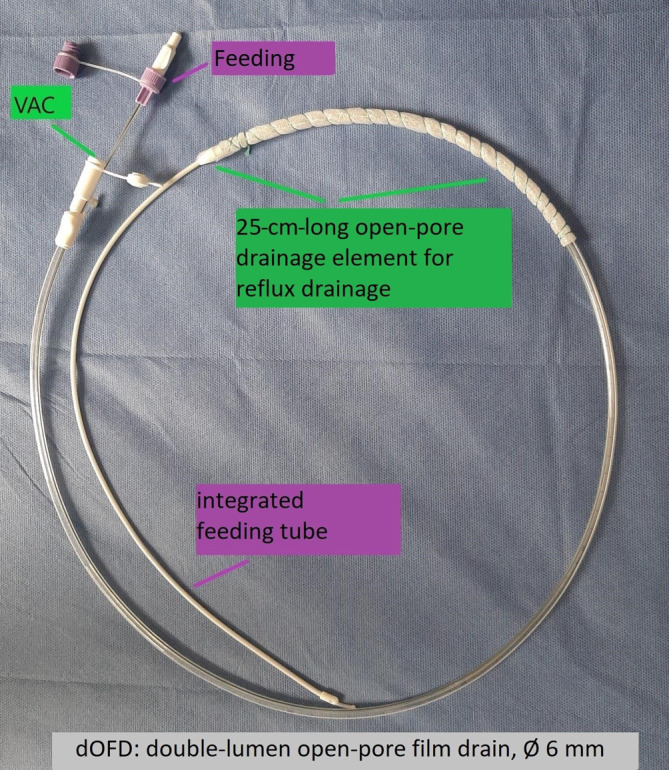
Double-lumen open-pore film drain. The distal end of the gastric segment of a Trelumina probe (Freka® Trelumina, Fresenius) coated with the open-pore drainage film (Suprasorb® CNP Drainage Film, Lohmann & Rauscher). Negative pressure (*VAC*) is applied to the gastric segment, allowing for continuous active suction and decompression of the stomach

**Fig. 3:**
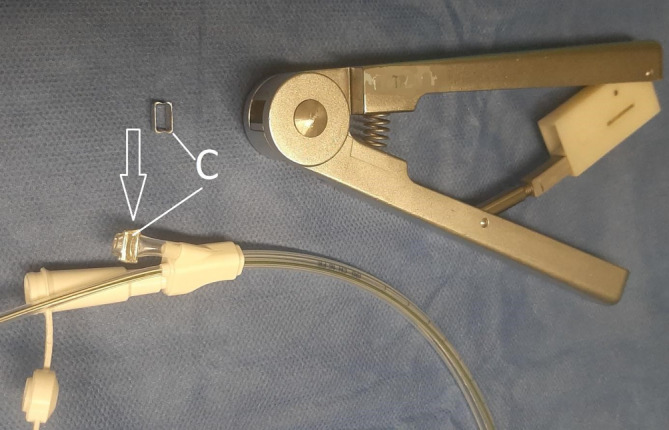
The ventilation channel of the Trelumina probe is occluded with a press clamp and its function is thus disabled. Press clamp (*C*) with pliers

A thin double-lumen open-pore film drain (dOFD) with an integrated feeding tube is used for PARD. This is made by covering the gastric segment of a Trelumina probe (Freka®Trelumina, Fresenius Kabi Deutschland GmbH, Bad Homburg, Germany) with a double-layered open-pore film (Suprasorb®CNP Drainagefolie, Lohmann & Rauscher International GmbH & Co. KG, Rengsdorf, Germany) over a length of 25 cm. A single layer of film should be used so that the diameter of the Trelumina probe is not significantly increased. Depending on the width of the film strip used, multiple layers may be applied in some places. This does not affect function, but it does increase the diameter of the probe segment. The ventilation channel of the Trelumina probe is occluded with a press clamp and its function is thus disabled. Two channels of the Trelumina probe are used in PARD (film-coated gastric drainage channel and the feeding channel). The dOFD has a diameter of only 6 mm (Figs. 2 and 3).

Intraoperatively, once the anastomosis is completed, the dOFD is inserted transnasally, using the same technique as for an NGT [13]. The dOFD is in effect a substitute for an NGT.

**Fig. 4:**
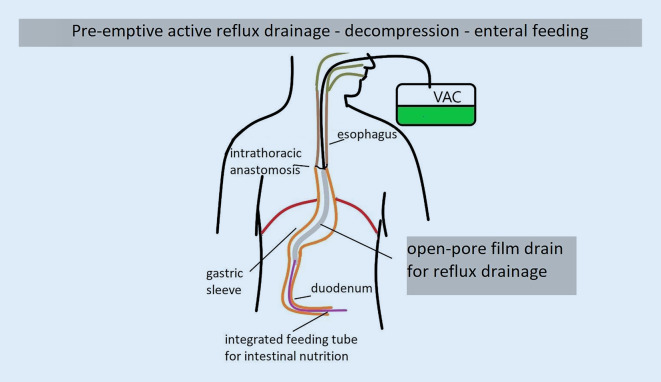
PARD method: In pre-emptive active reflux drainage (PARD), the thin open-pore film drainage tube is inserted transnasally using the same technique as for a nasogastric tube. The drainage element of the double-lumen open-pore film drain lies distal to the intrathoracic anastomosis in the gastric sleeve. Reflux secretions are suctioned using an electronic negative-pressure pump (*VAC*). The gastric sleeve is completely emptied and the anastomotic region is decompressed simultaneously. The integrated feeding tube lies in the jejunum. It can be used to give enteral nutrition during PARD

Under endoscopic guidance, the 25-cm-long film-coated drainage element (FDE) is positioned in the gastric sleeve. The proximal end of the FDE lies distal to the anastomosis. The FDE extends along the entire length of the gastric sleeve. The distal end usually extends to the prepyloric antrum or even through the pylorus with the tip lying in the proximal duodenum. The integrated enteral feeding tube (iT) is advanced deep into the jejunum via the duodenum. Using an electronic negative-pressure pump (ACTIV.A.C.; KCI, San Antonio, TX, USA), a continuous negative pressure of −125 mm Hg is applied to the FDE. This continuously aspirates and decompresses the stomach. Enteral nutrition with tube feeds is provided simultaneously via the iT. Water is given on the first postoperative day and then increased to tube feeds on the second and third days. No negative pressure is applied to the anastomosis (Fig. 4).

**Fig. 5:**
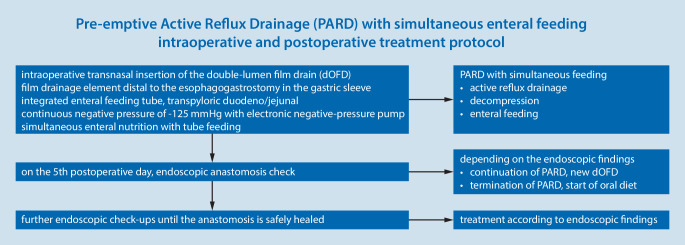
Pre-emptive active reflux drainage (*PARD*) treatment protocol: overview of the procedure

Our protocol (Fig. 5) is to routinely perform an endoscopic inspection of the anastomosis at 5 days postoperatively. Depending on the findings, PARD is then continued or stopped. If the wound is clean, PARD is stopped and oral feeding is started. If an at-risk anastomosis (ARA) is present, PARD is continued. The dOFD is changed every 3–4 days until the endoscopic findings confirm that the anastomosis is functioning properly. In any event, we also carry out further endoscopic follow-ups after completion of PARD to ensure that it has fully healed. All patients are given double-dose intravenous antacid medication postoperatively.

We define anastomoses as ARA if the following endoscopic signs of impaired wound healing are present at the first postoperative follow-up: widespread ulceration, signs of ischemia, necrosis, exposed clips, or visible sutures (Table 2).

**Table 2:** At-risk anastomoses

Endoscopic criteria for at-risk anastomoses
Exposed clips
Visible sutures
Widespread ulceration
Necrosis
Signs of ischemia

## Results

During the study period (November 2017–April 2023), PARD was used for all patients (*n* = 43) (age range: 53–80 years, median = 66 years; 35 male, 8 female) undergoing ILE. In total, 41 patients underwent open surgery and two underwent hybrid surgery; 36 of 43 patients received neoadjuvant therapy.

Endoscopic placement of the dOFD was performed intraoperatively after completion of the esophagogastrostomy. Simultaneously with the application of negative pressure, enteral nutrition was started in the early postoperative period via the integrated feeding tube.

The first endoscopic anastomosis follow-up was performed after a median of 4 days (2–7). All follow-up endoscopies revealed an empty and decompressed gastric sleeve. The anastomoses were free from reflux digestive secretions. Endoscopic visualization showed the anastomoses to be whitish and not greenish in color. No aspiration pneumonia was observed in any patient on PARD.

In 20 of 43 patients (47%), we saw disturbances in anastomotic healing, which we defined as ARAs (Table 2). In these patients, PARD was continued until endoscopic findings showed successful anastomotic healing. No repeat surgeries on the anastomoses were required. Other endoscopic interventions, such as ENPT with polyurethane foam drainage and direct negative pressure at the anastomosis site, or treatment with self-expanding or vacuum stents, were not required. Six of the 20 patients with an ARA (30%) developed a postoperative anastomotic stenosis (14% of the total cohort). This was easily treated with balloon dilatation. One patient died of pneumonia 18 days after surgery.

Healing was observed in all anastomoses (100%) treated with PARD. The median duration of PARD was 8 days (4–21; Table 3).

**Table 3:** Overview of all patients (*n* = 43) undergoing Ivor–Lewis esophagectomy in the period November 2017–April 2023. Since 2017, all of these patients have been treated pre-emptively with PARD at Marienkrankenhaus Hamburg^a^

Patient	Gender	Age (years)	Neo-adjuvant therapy	Tumor	Anastomosis in cm (from dental arch)	Insertion of dOFD (*n*)	First endoscopy (postop. day)	Duration of ENPT (days)	Healing of anastomosis	At-risk anastomosis (ARA)	Additional therapy on the anastomosis?	Stenosis	Dilatation
1	m	75	FLOT	ypT3 yPN1	30	1	7	7	+	–	–	–	–
2	m	53	FLOT	ypT3,ypN1	29	2	4	11	+	ARA	–	–	–
3	m	75	FLOT	ypT2, pN0	25	2	6	10	+	ARA	–	–	–
4	f	53	FLOT	ypT0, ypN0	15	2	4	8	+	–	–	–	–
5	m	63	–	pT1,pN0	25	1	4	4	+	–	–	–	–
6	f	72	–	pT1,pN0	19	1	4	8	+	–	–	–	–
7	m	62	FLOT	ypT3ypN1	25	7	2	21	+	ARA	–	+	+
8	m	73	FLOT	ypT2,pN0	25	6	2	19	+	ARA	–	–	–
9	f	79	CROSS	ypT0 pN0	23	2	4	7	+	ARA	–	–	–
10	f	61	FLOT	ypT3 pN1	23	2	3	10	+	–	–	–	–
11	m	67	FLOT	ypT3 yPN2	25	2	3	8	+	ARA	–	–	–
12	m	64	FLOT	ypT0 pN0	25	4	4	17	+	–	–	–	–
13	m	78	–	pT2 pN1	25	2	3	8	+	–	–	–	–
14	m	64	–	pT3 pN2	25	2	4	7	+	–	–	–	–
15	m	77	FLOT	ypT3 pN1	24	4	4	10	+	ARA	–	–	–
16	f	66	–	pT1b pN0	22	2	6	10	+	ARA	–	+	+
17	m	59	FLOT	ypT3 pN1	26	2	2	7	+	–	–	–	–
18	m	61	FLOT	ypT0 pN0	27	1	4	4	+	–	–	–	–
19	f	75	FLOT	ypT3 pN2	25	4	4	15	+	–	–	–	–
20	m	68	FLOT	ypT3 pN0	25	5	4	15	+	ARA	–	–	–
21	m	68	FLOT	ypT2 pN1	25	2	3	8	+	–	–	–	–
22	m	77	FLOT	ypT3 pN0	25	5	1	13	+	ARA	–	+	+
23	m	72	FLOT	ypT0 pN0	24	2	4	7	+	–	–	–	–
24	m	64	–	pT3 pN0	24	2	4	7	+	–	–	–	–
25	f	67	–	pT1b pN0	24	2	2	5	+	ARA	–	–	–
26	m	65	FLOT	ypT0 pN0	25	4	1	14	+	ARA	–	+	+
27	m	60	FLOT	ypT3 pN0	26	1	5	5	+	ARA	–	+	+
28	m	58	FLOT	ypT3 pN3	25	1	4	4	+	–	–	–	–
29	m	80	FLOT	ypT3 pN1	24	2	1	7	+	–	–	–	–
30	m	73	FLOT	yPT0 pN0	25	1	4	4	+	–	–	–	–
31	m	65	FLOT	ypT3 pN2	25	3	4	12	+	ARA	–	–	–
32	m	83	–	pT1 pN0	28	3	2	10	+	–	–	–	–
33	m	51	FLOT	ypT0 pN0	25	3	5	12	+	ARA	–	+	+
34	m	49	FLOT	ypT2 pN0	25	1	4	4	+	–	–	–	–
35	m	60	–	pT1b pN0	25	1	6	6	+	–	–	–	–
36	m	64	FLOT	ypT3 pN3	25	2	2	5	+	–	–	–	–
37	m	63	FLOT	ypT3 ypN1	25	2	5	8	+	ARA	–	–	–
38	m	70	CROSS	ypT0 ypN1	25	4	5	18	+	ARA	–	–	–
39	m	61	CROSS	ypT3 ypN1	29	2	2	6	+	–	–	–	–
40	f	66	–	pT1b pN0	25	1	7	12	+	ARA	–	–	–
41	m	74	FLOT	ypT1b ypN1	25	1	3	7	+	–	–	–	–
42	m	59	FLOT	ypT3 ypN1	25	3	3	12	+	ARA	–	–	–
43	m	79	FLOT	ypT1a ypN0	20	3	5	11	+	ARA	–	–	–
	*35 m; 8 f*	*M* *=* *66 (53–80)*			*M* *=* *25* *cm*	*M* *=* *2*	*M* *=* *4*	*M* *=* *8*	*Healing 43/43* *=* *100%*	*n* *=* *20*		*n* *=* *6*	*n* *= 6*

^a^The anastomosis healing rate is 100%. No repeat surgeries or other endoscopic interventions were necessary during the healing phase. One patient died of pneumonia 18 days after surgery

*f *Female, *m* male, *M* mean

## Discussion

Abdomino-thoracic esophageal resection has one of the highest morbidity rates in oncological surgery. Even with careful adherence to well-known strategies for preventing complications [2], there is a significant risk of developing clinically manifest anastomotic insufficiency. This healing disorder poses the greatest risk to patients.

In recent years, considerable progress has been made in the diagnosis of complications. Endoscopic inspection of the suture site is the most important procedure for excluding or confirming a healing disorder. Direct visualization enables the cause to be identified, and the size and blood flow of the anastomosis can be described precisely. Ideally, the necessary endoscopic therapeutic interventions to prevent infection can be performed immediately after diagnosis as part of the same examination. Endoscopy is complemented by computed tomography, which can provide additional information regarding extraluminal thoracic conditions [15].

There has been a clear shift in the surgical treatment of anastomotic healing disorders. Surgical approaches such as discontinuity resection with evacuation of the proximal esophagus as a salivary fistula and blind closure of the draining stomach are now extremely rare. The main focus of treatment is on emergency endoscopic interventional procedures, which are initially used to control or prevent mediastinitis.

Endoscopic negative-pressure therapy, which can be considered the therapy of choice in our region today, is particularly important in this context. The treatment success rate is approximately 85%. In most cases, anastomotic insufficiencies can be treated endoscopically and revision surgery can be avoided. In recent years, knowledge and mastery of this endoscopic intervention have increased and it has become an integral part of complication management after esophageal surgery, representing an important prerequisite in terms of making a high-risk surgical procedure very safe for patients [15].

It is striking that modern endoscopic techniques are driven by surgical departments that possess the surgical and endoscopic expertise to manage complications [16]. The development of ENPT on the upper gastrointestinal tract has been largely initiated by the surgical endoscopy working group at Marienkrankenhaus since 2006. In the interim, over 50 publications have reported on this diverse form of therapy for the upper gastrointestinal tract in numerous original case reports, retrospective case series, video publications, and further developments of technical materials (www. Endoscopicvacuumtherapy.com). The method of PARD described here has been developed based on the experience of numerous clinical applications of ENPT.

Initially, ENPT was administered to treat severe complications once they had occurred. In 2014, we reported on the use of ENPT to treat esophageal complications in a cohort of 35 patients. The insufficiency rate in our cohort was 17% at that time [17]. With the further development of drainage and placement techniques, the realization that duodenal reflux secretions can be optimally drained using ENPT and the inauguration of the intraluminal variant of ENPT [13], we gradually developed the concept of PARD. The use of the thin open-pore drainage film (Suprasorb® CNP Drainage Film, Lohmann & Rauscher) led to the development of an innovative thin negative-pressure drain (OFD) for ENPT that can be used like an NGT [18].

In simple terms, the OFD is an NGT to which a negative pressure can be applied. It differs from an NGT in that it empties the stomach completely, which is not the case with a passive NGT. With passive drains such as NGTs, drainage occurs by gravity and capillary pressure where there is positive pressure. In numerous endoscopic anastomotic follow-ups we have observed that the anastomotic region is flooded with reflux secretions, despite a correctly positioned passive NGT. The phenomenon of incomplete gastric emptying in the presence of an NGT will be readily confirmed by endoscopists examining these patients.

Our treatment algorithm provides for an early endoscopic check-up after 5 days. In some patients, this was not the case. A reason for an early check-up can be signs of infection. Longer probe insertions were tolerated in individual cases where there was a lack of endoscopy capacity or where a change would have been scheduled for a weekend. If reflux secretions can be aspirated, it is safe to assume that the dOFD is working properly. Initially, we never encountered insufficient suction. But if no reflux secretions are obtained, it is important to consider that the probe may not be working properly, for example, if the film is blocked. This can only be checked and corrected by changing the probe. Other problems include failing to change full canisters or pump malfunction.

Knowledge of the risks for anastomotic healing and of the altered physiology of the new digestive tract have led to the further development of ENPT in the direction of a new, minimally invasive pre-emptive method. The treatment principle behind it is simple. The aggressive secretions of postoperative reflux are kept away from the esophagogastrostomy in the vulnerable early phase of wound healing. Postoperative reflux contains gastric, pancreatic, biliary, duodenal, and oral enzymatic secretions whose physiological function is digestion. Postoperative reflux induces esophagitis and increases the risk of pulmonary aspiration. Active emptying also results in decompression of the gastric sleeve and the anastomotic region. Simultaneously, the integrated feeding tube in the dOFD enables early enteral nutrition. Surgical placement of a jejunostomy, which carries its own risks, is unnecessary [19].

Since 2017, we have been using this safety technique for all patients undergoing abdomino-thoracic esophageal resection. Despite the change in chief physician in April 2023, the method will continue to be used by the new departmental management as part of robotic esophageal surgery. Since the introduction of PARD, the cure rate of anastomoses after esophagectomy at the Marienkrankenhaus is 100%. We have treated 43 patients pre-emptively since the method was introduced. Assuming an insufficiency rate of 17%, without the use of PARD we would have expected anastomotic insufficiency in approximately seven of the 43 patients. In almost half of our patients (47%) we observed a disturbance of wound healing for which we defined the criteria of an ARA. This number may seem high, but we believe it correlates with the expected anastomotic insufficiency rate if PARD were not used. We assume that a significant proportion of these high-risk patients would have developed clinically manifest anastomotic insufficiency. We consider the early postoperative visibility of clips and suture material in particular to be an important warning sign. Müller et al. have described the risk of postoperative anastomoses in a similar way; they attempt to describe the extent of the restriction even more finely [23]. Our risk definition is simpler and more practical (Table 2). If one of the criteria is met, PARD is continued [14]. With a view to maximizing safety, we accept that some patients may be overtreated, prolonging the PARD and leaving the nasal dOFD in place.

The rate of anastomotic stenosis can be explained in terms of the criteria for wound healing disorders in ARAs. It is not a consequence of prolonged PARD. According to our extensive experience of endoscopic anastomotic follow-ups, including at other sites such as the rectum, ARAs in particular are at increased risk of stenosis if ischemia has been observed. Patients with an ARA are followed up endoscopically for a longer postoperative follow-up period. All stenoses were easily treated.

Another important safety aspect of the method is the avoidance of early postoperative aspiration due to reflux, which cannot occur in the absence of secretions.

In principle, PARD could also be carried out using open-pore polyurethane foam-coated negative-pressure drains (OPD) instead of film-coated drains. However, this requires special endoscopic insertion techniques. One disadvantage of the OPD currently on the market is the large diameter of the drainage element and the lack of a feeding tube. The integrated intestinal probe of the dOFD allows feeding to commence very early in the postoperative period, simultaneously with negative-pressure treatment and without the need for a jejunostomy. We consider a minimal drainage diameter, which makes direct transnasal insertion possible, to be a major advantage in the handling of the dOFD. The diameter of the Trelumina probe will increase slightly due to the film coating; this cannot be avoided as it is prepared individually by hand. Occasionally we observed epistaxis during insertion and changes of the dOFD. There was never any need for ENT treatment. It can be assumed that film probes can be produced with an even smaller diameter if manufactured industrially. With single-lumen film probes, we can achieve probe diameters of 4 mm.

The PARD method is solely for providing intragastric negative pressure with the aim of removing gastric reflux. The anastomosis itself is not treated with negative pressure. This could easily be achieved by positioning the FDE more proximally or extending the FDE. Film-based drains adhere less firmly to the adjacent tissue under suction than an OPD.

The pre-emptive postoperative intraluminal application of ENPT with OPD for high-risk anastomoses was demonstrated by Neumann et al. in a small case series. All the anastomoses healed [21]. One animal study supports this finding. Despite the intraoperative anastomotic defect, the anastomoses healed under intraoperative ENPT with intraluminal placement of the OPD over the anastomotic region [22, 23]. Individual groups have since begun to clinically use pre-emptive OPD with the intraoperative application of negative pressure over the anastomosis. Similarly good results have been reported [20, 24]. When used for high-risk anastomoses in revision surgery, anastomotic insufficiencies could not be prevented, but the use of ENPT is still considered helpful [25]. Initial clinical results for the application of the polyurethane-coated self-expanding stents under pre-emptive indications [26] are also available. The first systematic meta-analysis evaluating the pre-emptive use of ENPT for other indications following gastrointestinal tract surgery has already been published [27]. The benefits of pre-emptive ENPT for rectal anastomoses are also being investigated [28].

**Fig. 6:**
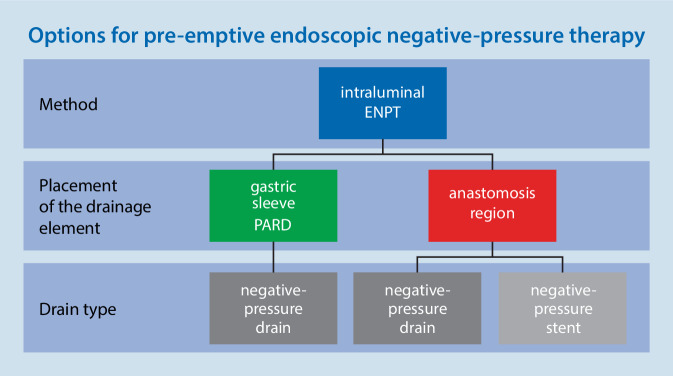
Illustration of the different types of pre-emptive intraluminal endoscopic negative-pressure therapy (*ENPT*). Treatment can be administered either in the gastric sleeve (pre-emptive active reflux drainage; *PARD*) or in the anastomotic area. It can be administered using negative-pressure drains or a negative-pressure stent. The open-pore drainage film or polyurethane foam can be used as drainage material in all cases. They can be used either with or without a feeding tube for simultaneous feeding

A 2015 study initiated to investigate whether intraluminal negative-pressure therapy with foam drains can reduce the rate of anastomotic insufficiencies could not be completed. A new study focusing on the same question has now been launched [29]. It would be of interest to prospectively compare the pre-emptive use of ENPT in a variety of possible settings. The following methods may be defined, but differ in terms of drainage material, the drain design, and the placement site. Options include intragastric reflux drainage alone (PARD), intragastric reflux drainage in combination with direct intra-esophageal negative-pressure application to the anastomotic region, and the application of negative pressure to the anastomotic region with a self-expanding negative-pressure stent (Fig. 6). All of these variants can use open-pore drainage films or polyurethane foam, with or without a feeding tube.

Of the various options, we consider PARD to be the simplest method from a technical point of view, with proven significant benefits. It is an integral part of our treatment protocol.

**Fig. 7:**
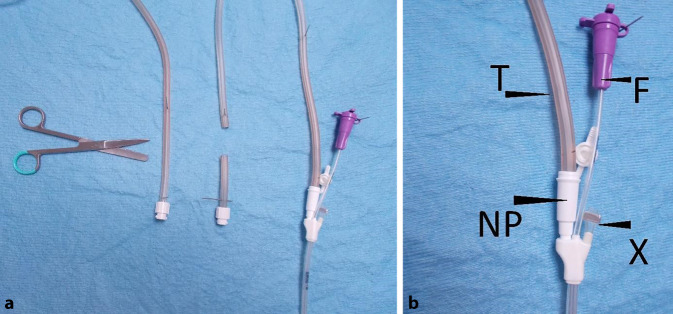
**a** Drainage tube of a reservoir canister of an ACTIV.A.C. pump KCI connected to the double-lumen open-pore film drain (dOFD). The connector on the drainage tube is snipped off. The cut end of the tube must be completely inserted into the attachment funnel of the dOFD. Negative pressure can then be applied. **b** Detailed photo shows the tube (*T*) inserted into the funnel (*NP*) and the ventilation segment (*X*) of the Trelumina probe modified for dOFD closed with a press clamp. The ventilation segment is closed because leakage would otherwise occur, and the pump would sound an alarm and not build up negative pressure. Feeding tube funnel (F)

To introduce the new method, preparations must be made not only for the procedure itself but also for postoperative care, as outlined below. Doctors and nursing staff in the operating room, intensive care, and general wards, as well as anesthesia and endoscopy departments, must be made aware. In our procedure, we have found it useful to place the probe after the actual surgery has been completed, with the patient still intubated and lying on the back. This can be done in the operating room or prior to extubation in the intensive care unit. The anesthetist needs to be aware that the anesthetic will have to be prolonged for 15–30 min due to the endoscopy. A negative-pressure pump with canister and the dOFD must be on hand. We make up the dOFD on the day before the procedure. After inserting the bite guard, a diagnostic gastroscope is used to look into the esophagus. We always use CO_2_ as the examination gas. The swift examination is limited to a careful general inspection. No mucosal fine diagnosis with gastric hyperinflation and distension is performed! Sudden movements should be avoided. Inversion in the gastric sleeve is not performed. The anastomosis is inspected, and blood flow is assessed. Changes such as edema or hematoma are often seen. There are usually some bloody secretions in the gastric sleeve, which are rinsed and drained. The pylorus is passed and the straightened duodenum is briefly inspected. The endoscope is removed. The guide wire of the feeding tube and the tube are lubricated. The dOFD is then introduced through one nostril and inserted into the cervical esophagus under endoscopic visualization. The passage of the tip of the dOFD over the anastomosis should be endoscopically guided. The probe is advanced further together with the endoscope using the usual endoscopic technique with assistance, and the probe should be secured to the nose when the endoscope is retracted. The feeding tube can be grasped with forceps and advanced through the pylorus. The FDE is placed far enough into the gastric sleeve so that it reaches from the anastomosis to the pylorus. The intention is not to cover the anastomosis, but it would be easy to do so. With the probe fixed manually to the nose, the gastroscope is removed and loop formation in the pharynx is ruled out. We secure the probe to the nasolabial fold with a suture to prevent it from accidentally becoming dislodged. The film-coated gastric drainage segment is connected to the negative-pressure pump tube and a suction of −125 mm Hg is applied. The guide wire of the feeding tube is removed and patency is checked. Water can be fed through the tube immediately after surgery. Routine clinical practice has shown that it is frequently difficult to connect the dOFD and the drainage tube. Staff responsible for postoperative care must be familiar with this simple procedure. The connection piece from the tube to the canister of the pump is snipped off and inserted into the attachment tube of the dOFD. This connection is sufficient, but it can be additionally secured with an adhesive dressing (Fig. 7). When the canister is full, it is changed. After approximately 5 days, anastomotic follow-up is carried out. We schedule the intervals between follow-ups so that they are integrated into the weekly endoscopy schedule.

A disadvantage of the PARD method, and of all other pre-emptive methods, is the fact that some patients might be overtreated and retain the nasal probe for a few days more than necessary. However, with PARD we also see two major advantages in these patients: firstly, the anastomoses heal with little inflammation in the early, vulnerable postoperative phase, and secondly, we reduce the risk of aspiration in the early postoperative phase by suctioning off reflux secretions.

We use the film-based drains in a much wider range of clinical settings in everyday clinical practice. Therapeutically, they expand considerably the spectrum of ENPT applications in numerous indications and replace or supplement foam-based drains (e.g., in small defect openings, fistulas, in the duodenum, urology; [30–32]). They can be used in all surgeries involving anastomoses in the upper gastrointestinal tract.

## Conclusion

Intrathoracic anastomoses are the Achilles heel in the early postoperative phase after Ivor–Lewis esophagectomy (ILE). Meticulous attention should be paid to anastomotic healing. Endoscopic negative-pressure therapy is a new tool that actively supports the local wound healing process at the site of the anastomosis. With the introduction of pre-emptive active reflux drainage (PARD) in ILE using a double-lumen open-pore film drain (dOFD) with integrated feeding tube, there was sufficient healing of intrathoracic anastomoses in all 43 patients. No repeat surgeries on the anastomosis or other endoscopic procedures were necessary. The technically simple method allows for enteral nutrition with simultaneous and continuous gastric emptying and decompression. Our results suggest that PARD has a strong protective effect on anastomotic healing and can reduce the risk of anastomotic insufficiency. The integrated feeding tube of the dOFD enables early postoperative enteral nutrition alongside the application of negative pressure.

## Abbreviations

AEG: Adenocarcinoma of the oesophagogastric junction
ARA: At-risk anastomoses
dOFD: Double-lumen open-pore film drain
ENPT: Endoscopic negative-pressure therapy
FDE: Film-coated drainage element
ILE: Ivor–Lewis esophagectomy
iT: Integrated enteral feeding tube
NGT: Nasogastric tube
OFD: Open-pore film drain
OPD: Open-pore polyurethane foam drain
PARD: Pre-emptive active reflux drainage
PR: Postoperative reflux
VAC: Negative pressure

## Declarations

This article does not contain any studies with human participants or animals performed by any of the authors.
